# Supplementary material for: Understanding the links between human health, ecosystem health, and food systems in Small Island Developing States using stakeholder-informed causal loop diagrams
Source: PLOS Glob Public Health. 2023 Sep 19;3(9):e0001988. doi: 10.1371/journal.pgph.0001988 (PMC10508617; doi:10.1371/journal.pgph.0001988)
Supplement: S1 Table — (DOCX) [file pgph.0001988.s001.docx]

| Label | Variables in the Pathway | Brief description |
| --- | --- | --- |
| R1 | → Political support for climate resilience and sustainable health → Conventional commercial interests → | Strong political support for climate and sustainable health reduces the impact of conventional commercial interests which are currently degrading climate and health. This in turn reduces their influence on political support for climate and health-sustainable policies. The loop can also work in the opposite direction (i.e. decreasing support increases the impact of commercial interests, increasing their influence and undermining support). |
| R2 | → Conventional commercial interests → Food damaging to planetary health → conventional commercial interests → | Conventional commercial interests are typically fossil fuel-based and degrade ecosystems, producing food that damages planetary health. The more this type of food is produced, the more commercial interests are reinforced through increasing profits and market share. |
| R3 | → Food damaging to planetary health → Agroecologically produced healthy food → | Food that is damaging to planetary health will compete for agroecologically produced healthy food. Where damaging food is produced, there will be less agroecologically produced food. The opposite is also true, where more agroecologically produced food is present, there will be less food damaging to planetary health. |
| R4 | → Agroecologically produced healthy food → Cohesive, unified, thriving communities → | Agroecologically produced healthy food reinforcing cohesive and thriving communities. Those communities, in turn, can reinforce the production of this kind of food if they choose. |
| R5 | → Cohesive, unified, thriving communities → Political support for climate resilience and sustainable health → Ecosystem health and resilience → agroecologically produced health food → | Cohesive communities can also build political support for climate resilient policies, improving ecosystems and supporting the production of agroecologically produced healthy foods. Further contributing to cohesive communities. |
| R6 | → Political support for climate resilience and sustainable health → Ecosystem health and resilience → Agroecologically produced healthy food → Healthy diets → Conventional commercial interests → | Strong political support for climate and sustainable health reduces the impact of conventional commercial interests which are currently degrading climate and health. This in turn improves ecosystem health and resilience and makes it possible to produce agroecological healthy foods. This contributes to healthy diets, reduces the influences of food damaging to planetary health, and the impact of commercial interests, further strengthening political support. |
| R7 | → Ecosystem health and resilience → Agroecologically produced healthy food → | A healthy ecosystem allows for production of agroecological food systems and reinforces healthy ecosystems and reducing the impact of food damaging to planetary health. |
| R8 | → Conventional commercial interests → Food damaging to planetary health → Healthy diet → | Conventional commercial interests produce food damaging to planetary health and are often undermining healthy diets. Consumption of these foods strengthens the conventional commercial interests to keep producing these foods. |
| R9 | → Cohesive, unified, thriving communities → Agroecologically produced healthy food → Healthy diet → Good physical and mental health → | Cohesive communities can engage in agroecologically producing healthy foods, this contributes to healthy diets, good physical health and good mental health. This reinforces cohesive communities. |
| R10 | → Political support for climate resilience and sustainable health → Cohesive, unified, thriving communities → | Cohesive communities can reinforce support for climate resilience and sustainable health where they see the value of engaging in sustainable practices. This political support can lead to policies that sustain cohesive communities living in a sustainable way. |
| R11 | → Healthy diet → Conventional commercial interests → Food damaging to planetary health → Agroecologically produced healthy food → | Healthy diets are often in competition with those produced by conventional commercial interests which are damaging to planetary health, producing fewer of those foods leaves more room for agroecologically produced healthy foods, further reinforcing healthy diets. |
| B1 | → Conventional commercial interests → Ecosystem health and resilience → | Conventional commercial interests undermine ecosystem health and resilience. That ecosystem health can then affect the long term capacity for commercial interests to produce food. |
