# Supplementary material for: Understanding the links between human health, ecosystem health, and food systems in Small Island Developing States using stakeholder-informed causal loop diagrams
Source: PLOS Glob Public Health. 2023 Sep 19;3(9):e0001988. doi: 10.1371/journal.pgph.0001988 (PMC10508617; doi:10.1371/journal.pgph.0001988)
Supplement: S2 Table — (DOCX) [file pgph.0001988.s002.docx]

| Label | Variables in the Pathway | Brief description |
| --- | --- | --- |
| R1 | → Improved population wellbeing and ecosystems → Community ownership → Resilience of the local food supply → | Strengthening the local food supply improves the population and ecosystem wellbeing, creating a sense of community ownership, further strengthening the resilience of the food supply. |
| R2 | → Co-creating community food intervention with community partners → Provision of needs and resources → Adaptive capacity → Resilience of the local food supply → Improved population wellbeing and ecosystems → Community ownership → | Co-creating community food interventions with community partners and ensuring the provision of needs and resources strengthens adaptive capacity, this reinforces the resilience of the local food supply, improving population wellbeing and strengthening community ownership. This then would strengthen participation in co-created interventions. |
| R3 | → Co-creating community food intervention with community partners → Social capital brough by community embedded partners → Skills and knowledge building → R2 → | Co-creating community food intervention with partners builds social capital for those partners, strengthening skills and knowledge building, and feeding into R2. |
| R4 | → Co-creating community food intervention with community partners → Monitoring and evaluation → Responsiveness to outcomes or circumstances → Adaptable learning from the project → Clear objectives → | Co-creating interventions includes monitoring and evaluation which improves the responsiveness to outcomes or circumstances, allows for adaptable learning, strengthening clear objectives and reinforcing interventions. |
| R5 | → Stable funding → Responsiveness to outcomes or circumstances → Adaptable learning from the project → Findings and dissemination → | Stable funding improves responsiveness to outcomes or circumstances leading to adaptable learning, more dissemination of findings, and reinforcing funding. |
