# Supplementary material for: Understanding the links between human health, ecosystem health, and food systems in Small Island Developing States using stakeholder-informed causal loop diagrams
Source: PLOS Glob Public Health. 2023 Sep 19;3(9):e0001988. doi: 10.1371/journal.pgph.0001988 (PMC10508617; doi:10.1371/journal.pgph.0001988)
Supplement: Attachment — Submitted filename: ResponsetoReviewers_30July2023.docx [file pgph.0001988.s003.docx]

**Manuscript title:** Understanding the links between human health, ecosystem health, and food systems in Small Island Developing States using stakeholder-informed causal loop diagrams

Each of the reviewers’ comments are reproduced below followed by the authors’ responses indicated by the symbol 🡺

**Responses to Reviewer 1**

Introduction is quite long - paragraphs should be clearly structured and could be a little shorter. Writing style needs editing throughout and arguments are sometimes difficult to follow.

🡺Thank you for this comment. We are grateful for the reviewer’s suggestions that follow on how to improve the text and in addition have simplified the text where possible. We are pleased to note, however, that the second reviewer described the manuscript as, ‘very well-written and very clear’.

Line 55 - SIDS do not contribute that much to greenhouse gas emissions - this should be noted. I.e. SIDS food systems feel the impacts of climate change, but do not contribute to it significantly

🡺We have noted that SIDS contribute less than 1% of global greenhouse gas emissions (figure from the United Nations Development Programme)

Line66 onwards: “Adapting food systems to support food security and sovereignty, reduce vulnerability to rapid environmental change and enhance productivity and equity has been a major policy objective in SIDS(9) with a recent commitment in the Caribbean, for example, to reduce the regional food import bill by 25% by 2025” - there are a lot of concepts in this sentence which need to be more tightly linked together, e.g. environmental degradation is not directly linked with food imports

🡺We have split this sentence in two and made clear that the goal of increasing food security and sovereignty is set within the context of vulnerability to natural disasters.

P84 “However, despite…: - I would start a new paragraph here. Also needs a clearer flow of argument/segue here as to why interventions targeting food security are related to climate change

🡺We have started a new paragraph. At the end of the preceding paragraph, we have added, “The cascading impacts of, and vulnerability to, climate change further complicates these food system interventions that seek to lessen the burden of malnutrition and further environmental degradation. Mitigation of climate change is clearly outside the control of SIDS, given their very low contribution to global greenhouse gas emissions. Adaptation to the effects of climate change is where their focus must lie, with challenges from extreme weather events, rising sea-levels, storm surges, coastal erosion and soil salinization, and declining health of coral reefs and fish stocks”.

Line 111: should this be pressing questions?

🡺Thank you, we have corrected this.

Line 121: “participatory complex systems thinking methods” needs a citation

🡺Two citations are given.

Methods
Line 127 onwards - what was the time course of the project?

🡺We have entered the dates of the ICoFaN project.

Would be good to include a table or more comprehensive list of the stakeholders involved

🡺We have described the backgrounds of the stakeholders from the SIDS settings, as well as from the UK and Canada.

Results
Overall well done. Would be good, where possible, to see where the results you present came from e.g. which stakeholders or which countries said what. What is there all makes sense but more explicit links to the context of your data would help highlight the primacy and novelty of your findings.

Line 214: I don’t think the term ‘actors’ works for ‘four major actors’ as you use ‘actors’ elsewhere with a different meaning- could you use ‘factors’?

🡺We hope that we have clarified the use of the term ‘actors’, firstly by noting that we ‘identify four major *categories of* actors’ in the ‘generalized system determining food production in SIDS’ and secondly by describing more precisely who we are referring to as ‘actors’. The title for table 1, for example, has been changed so that it is immediately clear that the actors described within the table are those relevant to intervention co-creation undertaken by communities and researchers.

Line 240 “Political actors..” Should be a new paragraph

🡺We have moved this to a more appropriate position in the text, with it now being the second part of the first paragraph under the sub-heading, ‘Political determinants……’.

Line 249 onwards: this section contains a lot of really good points but could be more clearly structured to link the key issues e.g. agrochemical use, fossil food use, etc. to the key food production processes e.g. conventional farming, import of ultra-processed foods etc. At the moment it is all too jumbled together and the way it is presented the information loses its rigour.

🡺We hope that we have made this section clearer by breaking it into two paragraphs and (where appropriate) by elaborating on the descriptions of the relationships.

Figure 2 - how is government commercial interests in unhealthy foods and agricultural practices dealt with?

🡺Thank you for this. In our framework, the strengthening of community engagement in local food systems is seen as reducing the influence of commercial interests (B1), which we have now mentioned in the text.

Discussion
Line 406 - virtual group model
building sessions when face-to-face meetings were severely limited during the SARS-Cov
pandemic. - is this information in Methods?

🡺Thank you for this question and spotting this omission. We have added this important detail to the methods section.

Discussion is a little short - would be good to have a little more detail on examples of practical application of figures 1 and 2, and/or best first steps for governments and other stakeholders

🡺Thanks, to help address this feedback we have added text describing how the framework has influenced the design of a recently funded project.

Conclusion section is good, however I don’t see the immediate logic in the argument of this sentence: Global food systems are second only to energy production as a source of anthropogenic greenhouse gas emissions, and the ensuing climate change is itself making adequate food production more difficult.” - could this include an example of what element of SIDS food systems is CONTRIBUTING to climate change?

🡺Thank you for this good point. We have added the following after that sentence: ‘. ‘Although the contribution to global greenhouse gas emissions from the SIDS is relatively small, a reliance on food imports generated by conventional farming practices and the associated need for shipping links SIDS to this damaging feedback loop. In addition, fossil-fuel driven conventional farming practices within SIDS contribute to the degradation of local ecosystems.’

**Responses to Reviewer 2**

This manuscript makes an important contribution to the field, namely by identifying and describing the causal interrelationships among agricultural practices, food systems, climate and other forms of environmental change, and human health in Small Island Developing States (SIDS). Understanding these causes and effects is critical to build climate resilience and effective, community-driven adaptation strategies, as well as to improve food security and human health status. Even though this manuscript presents the results of a theoretical exercise rather than data from actual SIDS case studies, it highlights key sectors and actors that affect each other and need to be addressed to improve conditions on SIDS. This manuscript is very well-written and very clear.

🡺Thank you for this summary of the manuscript’s content and importance.

Specific comments are listed below. Overall, they are minor with the exception of the observation that Figure 1 seems unnecessarily complex, and that Figure 2 contains “R”s that seem redundant with the clear relationships indicated with arrows.

Line-specific comments:

The long list of “interventions designed to adapt SIDS food systems” in lines 74-82 is important information, and it would be helpful to contextualize the results of their study if the authors explained these specific interventions more, have a few examples, and perhaps discussed briefly why they are insufficient (because the authors state that despite these interventions, “SIDS food systems remain highly vulnerable…”).

🡺We have added to the paragraph that follows this list, and hope that the argument is made more convincingly that the challenges to SIDS and their food systems from climate change are multi-faceted, and that interventions to date have failed to properly address this.

Lines 184-5: The authors list only one “indicator of food security (increasing dependence on food imports).” 1) This is actually an indicator of food insecurity, and 2) it should probably be acknowledged that there are also other indicators of food security.

🡺Thank you for this comment, which prompted us to reflect that all three dimensions of the central reference mode described in that paragraph can be seen as aspects of food insecurity. In particular, they align with the recent proposal from Clapp and colleagues (2022) to broaden the definition of food security from the well-known ‘four pillars’ to also include sustainability and agency. We had added a sentence to this effect at the end of this paragraph.

Lines 209-211 and Figure 1: This figure is a bit complex, and the placements of “R”s and “B”s doesn’t always clearly indicate what relationship is being referred to. For example, why is “R8” listed where it is? And “R7” refers to two distinct relationships. (?) It also seems that there are both beneficial and harmful causal pathways, both in terms of the arrows used in the diagram and the “R”s, so could they be distinguished in this figure? Also, the term “ecosystem damaging unhealthy food” is misleading – rather than refer to “unhealthy food,” “agricultural practices” or “food system” is what is “ecosystem damaging.” I make further comments about Figure 1 in several comments below.

🡺We have added a legend to each of the figures to help address this comment and changed the term in Figure 1 to ‘food damaging to planetary health’ (i.e. both to the health of human populations and the ecosystems on which they depend*).* We have added two Supplementary Tables describing the pathways for each figure. These tables enable a reader to trace each of the feedback loops, allowing them to double check, should they wish to, that they are reading figures correctly.

Line 215: Regarding “agroecological food interests” – this is the first time agroecology is mentioned, but this comment relates to the entire paper. It would be useful to clarify if by this term they mean any form of agricultural practice or food system that is not “conventional commercial.” Some people use the term “agroecology” in a general way to mean sustainable agricultural practices, but it can be defined in several ways, and an agroecological approach may involve any of numerous distinct farming methods. Best for the authors to clarify how they are using this term, because in this paper, “agroecology” is the sole alternative to “conventional commercial interests” and “ecosystem damaging unhealthy food” (see Figure 1). Many alternatives could be included.

🡺Thank you for this important comment. We hope that we have given greater clarity as to how we are using the term ‘agroecology’ by providing a definition and description at the end of the methods section. We also note here that other terms to denote ‘sustainable’ approaches to food production may be used and indicate why we prefer the term ‘agroecology’.

Line 231: I would put a paragraph break here at “A climate supportive political…” In general, it would be easier to read and keep track of the explanations of the “R” feedbacks if shorter paragraphs are used.

🡺We have put in a paragraph break.

Lines 241-247: This description from Fiji is an example of the relationship between politics and commerce and thus could be moved to line 231 (before the paragraph break suggested here).

🡺Thank you. We have done this.

Line 255: The subject word “They” needs to be defined for clarity.

🡺Word ‘they’ replaced with ‘commercial actors’.

Line 256: I recommend a paragraph break here as well, at the end of the line.

🡺Paragraph break entered.

Lines 258 and Line 284 (and Figure 1): It seems a bit confusing why “R7” refers to two distinct feedback relationships. Can this be clarified?

🡺This was an error, and in line 258, R7 has been replaced by B1. Thank you for spotting this.

Line 262: The “presence of pesticides” does not only “undermine healthy diets” and “damage the health of farmers” but also damages the health of food consumers, damages ecosystem health, contaminates water bodies, may destroy plants, and exposes various wildlife to potentially damaging toxic effects. Also “healthy diets” is a complex term that may be used even if conventionally grown foods (those that may have pesticide residues) are included. For example, one can define a “healthy diet” as full of fruits and vegetables, regardless of if they are grown conventionally or organically.

🡺We have explicitly noted the potential for pesticide residues and put ‘healthy diets’ in inverted commas – which we hope addressed this comment.

Line 266: “imported foods” are not necessarily of higher quality than “local agricultural products,” and may be more likely to contain conventionally-grown or processed food ingredients, pesticide residues, and other contaminants. This should be clarified.

Line 268: What specific sorts of “regulation” should be enacted to control “commercial producers” should be explained here.

🡺For the above two comments, we have made explicit that the assumption is made that imported foods come from better regulated settings and are thus less likely to contain high levels of pesticide residue.

Line 272: This is a good place to provide more detailed definition and explanation of what the authors mean by “agroecology” here. (See also comment above regarding line 215.)

🡺As noted above, we have addressed this in the methods section.

Lines 279-281: It would be helpful to give a specific example of “agroecological food production” that illustrates how it is a beneficial alternative to “conventional monocrop food production practices in SIDS.”

🡺We have added in a sentence providing some examples of how ‘agroecological approaches’ may achieve greater sustainability.

Line 285: It would be helpful to define what the authors mean by “community-based principles.”

🡺We have expanded this sentence to say, ‘including local ownership and agency’.

Line 286: Before saying that agroecology may “reinforce healthy diets,” first it should be stated that agroecology may produce better health outcomes driven by healthier diets, then say these diets may be reinforced.

🡺Thank you – done.

Lines 290-302: This paragraph describes an important scenario whereby community cohesion leads to better food systems, but it is not a “given” that a community would be unified in its focus on local food productions, which approaches to food and farming would be chosen, and whether the entire community would support the same policies. If a “cohesive” approach is needed, it might be best to acknowledge that this “cohesion” probably would need to be cultivated, and how would that best be done?

🡺Thank you for this comment, we agree and have added new text to better address this important issue.

(Line 303) and Figure 1: Why are B1 and B2 not explained?

🡺B1 is now further explained in the text as well as being detailed in supplementary table 1. B2 has become R9, and the pathway is also detailed in supplementary table 1. It was our error in the initial submission to have described this loop as a ‘balancing’, when the overall direction of the relationships means that it is ‘reinforcing’.

Line 317 and Table 1: Clarify “how we define a community” – technically, isn’t this more about defining or identifying ideal roles of community members? And what is ideal may not necessarily be present in practice.

🡺We have revised the text to address this comment. Thank you.

Line 319 and Table 1: For the “Role” of “Community members,” it is certainly desirable to have them be “co-creators and implementers of the intervention” but this isn’t necessarily always going to happen in all cases, right? Should this be clarified? Also, the second bullet point under “Description” of “Community members” is very specific – why is this intervention listed but not others?

🡺We agree with the point being raised here and have revised the text accordingly.

Table 1 (continued): For the “Role” of the “Researcher,” the description makes it sound like “Community members” are not necessarily going to be creating interventions but that they may be imposed on them by “experts.” This wording should be clarified because it potentially contradicts the “Role” of “Community members.”

🡺We agree and have edited the text to address this concern.

Table 1 (continued): For the “Description” of the “Funder,” it seems likely that funders should be responsive not only to researchers but also to community leaders and/or community-embedded partners, those actually implementing interventions.

🡺We agree and have added this to the text, thank you.

Line 326: Indent?

🡺Corrected.

Line 327: Shouldn’t “relationship” be plural?

🡺Thank you – corrected.

Lines 330-1: Again, to clarify how agroecology is being conceived of here, it would be very helpful to give an example of how “implicit in that resilience is the adoption of agroecological principles.”

🡺We have edited this text and further elaborated on the principles as requested.

Figure 2: I’m not sure why the “R” feedback relationships are needed in Figure 2. The relationships are clearly described using text, and the authors don’t really describe these “R”s as distinct from the relationships indicated by arrows. This seems like different figure content than that in Figure 1.

🡺We have added a legend to both Figures to help with clarity. Identifying these “R” and “B” loops is an important part of the method and analysis. As noted above, we have also provided to two supplementary tables that list the variables in the individual loops and give a brief narrative summary of each loop’s effects.

Line 376: “policy and political” is awkward wording here. Seems like the authors could clarify that these are elected officials and decision-makers.

🡺We have clarified the text as advised, thank you.

Line 399: Vulnerabilities of “children, adolescent girls, women and seniors” is not noted or described elsewhere, so it should not be brought up here without more description. It is an important point, but best not to casually bring it up in the conclusion without it being explained earlier. Also, it is not a given that attention to all the relationships in Figures 1 and 2, both positive and negative, will necessarily prioritize those most vulnerable. Focus on vulnerabilities, age and gender equity, etc. must be deliberate and cultivated through the use of specific equity lenses.

🡺We have removed the general reference to these potentially vulnerable groups.

Lines 405-7: I am not an expert on the methods used here, so it would be helpful if the authors could explain how their approach “places control of the causal mapping firmly in the hands of the stakeholders.” How does that work? And how is it “flexible”?

🡺We have further elaborated to address this comment.

In several places, semi-colon usage is incorrect: line 58, list in lines 74-82, lines 307-8, line 311

🡺Thank you – we have made these corrections.
